# Short exposure to an enriched environment accelerates plasticity in the barrel cortex of adult rats

**DOI:** 10.1016/j.neuroscience.2006.02.043

**Published:** 2006

**Authors:** V. Rema, M. Armstrong-James, N. Jenkinson, F.F. Ebner

**Affiliations:** a National Brain Research Centre, Nainwal Mode, Manesar, Haryana 122050, India; b Department of Psychology, Vanderbilt University, Nashville, TN 37240, USA; c University Laboratory of Physiology, Parks Road, Oxford OX1 3PT, UK

**Keywords:** experience-dependent modifications, use-dependent plasticity, enriched environment, whisker-pairing, deprivation, receptive field changes, DC, D cut whisker, DP, D paired whisker, EE, enriched environment, EEWP, enriched environment whisker-paired, LD, light/dark, LTD, long-term depression, LTP, long-term potentiation, NMDA, *N*-methyl-d-aspartate, PSTHs, post-stimulus time histograms, SC, standard cage, SCWP, standard cage whisker-paired, S.E.M., standard error of the mean, SG, supragranular layer, MWU, Mann-Whitney *U*, WMPSR, Wilcoxon matched pair sign rank, WP, whisker-pairing

## Abstract

Cortical sensory neurons adapt their response properties to use and disuse of peripheral receptors in their receptive field. Changes in synaptic strength can be generated in cortex by simply altering the balance of input activity, so that a persistent bias in activity levels modifies cortical receptive field properties. Such activity-dependent plasticity in cortical cell responses occurs in rat cortex when all but two whiskers are trimmed for a period of time at any age. The up-regulation of evoked responses to the intact whiskers is first seen within 24 h in the supragranular layers [Diamond ME, Huang W, Ebner FF (1994) Laminar comparison of somatosensory cortical plasticity. Science 265(5180):1885–1888] and continues until a new stable state is achieved [Diamond ME, Armstrong-James M, Ebner FF (1993) Experience-dependent plasticity in adult rat barrel cortex. Proc Natl Acad Sci U S A 90(5):2082–2086; Armstrong-James M, Diamond ME, Ebner FF (1994) An innocuous bias in whisker use in adult rat modifies receptive fields of barrel cortex neurons. J Neurosci 14:6978–6991]. These and many other results suggest that activity-dependent changes in cortical cell responses have an accumulation threshold that can be achieved more quickly by increasing the spike rate arising from the active region of the receptive field. Here we test the hypothesis that the rate of neuronal response change can be accelerated by placing the animals in a high activity environment after whisker trimming. Test stimuli reveal an highly significant receptive field bias in response to intact and trimmed whiskers in layer IV as well as in layers II–III neurons in only 15 h after whisker trimming. Layer IV barrel cells fail to show plasticity after 15–24 h in a standard cage environment, but produce a response bias when activity is elevated by the enriched environment. We conclude that elevated activity achieves the threshold for response modification more quickly, and this, in turn, accelerates the rate of receptive field plasticity.

It is well established that modifications of cortical neuron responses occur after peripheral nerve damage (Merzenich et al., 1983) and injuries to spinal cord (Jain et al., 2000). However, changes in the normal usage of the peripheral receptors without any neural injury also can induce significant receptive field changes in cortex. For example, in the kitten visual system monocular experience leads to increased cortical response to inputs from an open eye and decreased response to inputs from a lid sutured eye (Hubel and Wiesel, 1963). In adult monkeys, the area of the used finger representation can be increased in somatic sensory cortex after prolonged training on frequency discrimination tasks using the same distal digit pads every day (Recanzone et al., 1992).

Neurons in the barrel cortex of adult rats also exhibit activity-dependent plasticity after whisker experience is altered by trimming all but two whiskers on one side of the face (a.k.a. “whisker-pairing” or WP) (Diamond et al., 1993; Armstrong-James et al., 1994). The changes in cortical receptive field characteristics share many of the properties known to be important for producing long-term synaptic potentiation, such as a dependence on *N*-methyl-d-aspartate NMDA receptor activation (Rema et al., 1999) and intact cholinergic mechanisms (Sachdev et al., 1998). Barrel column neurons in all layers tested show enhanced responses to the two intact whiskers and a depression of responses to stimulation of the trimmed whiskers (Armstrong-James et al., 1994). However, the time course for generating this Hebbian type of sensory plasticity is not identical for neurons in all cortical layers. For rats in standard laboratory plastic cage environments 24 h of whisker trimming is adequate to produce plasticity of layer III neurons and to a lesser extent infragranular layers, but inadequate to induce changes in layer IV barrel cells (Diamond et al., 1994). After 3 days of WP experience, neurons in all layers show modified responses, which persist for as long as the whiskers are trimmed (Armstrong-James et al., 1994). Cortical receptive field modification induced by a bias in activity levels arising from trimmed and untrimmed whiskers is, therefore, a form of experience-dependent plasticity.

We wanted to determine whether these activity-dependent events would be accelerated by exposure to an enriched environment (EE), which has been shown under a variety of conditions to accelerate cortical plasticity (Wallace et al., 1992; Rema and Ebner, 1999; Piche et al., 2004). Our test was to record cortical cell responses after a short period (15 h) of increased sensory challenge that is clearly less than the period required to produce significant receptive field changes when the animals are housed two to a standard plastic cage. Our results indicate that the rate of response modification in primary sensory cortex can be accelerated by environmental conditions. The very shortest period required for experience-dependent plasticity cannot be determined with the present experimental design because the whisker trimming, enriched experience, and recording would have to be sequenced without using drugs, which requires an awake animal preparation.

## Experimental procedures

All the experiments carried out for this study were in compliance with procedures approved by the Vanderbilt University Institutional Animal Care and Use Committee and followed NIH guidelines for use of animals in research. Adult, male Long Evans rats, 2–3 months of age, were used in these experiments. The number of animals used for this study was the minimum required, and care was taken to minimize their suffering.

### Experimental groups

A control group of adult rats was kept in standard (8-inch×10-inch×18-inch) laboratory plastic cages, housed with one other rat. This condition was designated as standard cage non-whisker-paired or “SC” rats (*n*=5). A second group of (SC) rats was whisker-trimmed for 15 h prior to recording cortical cell responses. The whiskers were trimmed at 6:00 PM in the evening and the animal was anesthetized with urethane at 9:00 AM the next morning for electrophysiological recording. This group was called “SCWP” rats (*n*=6). Two other groups of rats were exposed to an EE. One group had all whiskers intact, termed the EE group, while the other group was whisker-paired for 15 h, called “EEWP” animals (*n*=6 in both groups).

The EE consisted of a large (36-inch×36-inch×30-inch) cage made of 1/2-inch wire mesh with ladders to three platforms that rendered movement in the environment highly three-dimensional. Novel objects of various sizes and shapes were placed in the bottom of the cage, and changed every day, while food was available on the top level and water bottles with sipping tubes protruded into the enclosure. Six rats were introduced into the cage for 12 h during the dark phase of a 12-h light/dark (LD) cycle for 2 successive 12 h dark periods. During the light (low activity) phase of the 12-h LD cycle the EE rats were returned to a standard laboratory plastic cage (two rats in each cage). EE exposure during the first night was to acclimate the animals to the novel environment and companions. The following day at 6:00 PM one of the rats had all but D1 and D2 whiskers trimmed on the right side close to the face (whisker-paired) before being placed in the EE. At 9:00 AM the following day the EEWP rat or a non-whisker-paired EE rat was analyzed under urethane anesthesia.

### Analysis of plasticity

To simple observation the animals appeared to use the intact, pair of whiskers to palpate, explore, and “whisk” in a manner similar to when all the whiskers were intact, although asymmetry of bilateral whisker movements has been reported after unilateral whisker pairing (Sellien et al., 2005). Detailed procedures used for inducing and analyzing WP plasticity are described in Armstrong-James et al. (1994) and in Rema et al. (1998).

### Electrophysiology

Electrophysiological analysis was carried out under urethane anesthesia (1.5 g/kg body weight, i.p.). The rats were positioned in a stereotaxic apparatus with body temperature maintained at 36–37 °C using a rectal thermistor coupled to an electronically controlled heating pad. Barrel cortex contralateral to the trimmed whiskers (left hemisphere) was exposed for recording. No recordings were made from the hemisphere ipsilateral to the whisker trimming because of the well-documented interhemispheric disturbances that occur after altering activity levels in one hemisphere (Li et al., 2005; Li and Ebner, 2005).

Carbon fiber microelectrodes (Armstrong-James and Millar, 1979) were used to record single unit action potentials. The position of the penetrations was controlled with an accuracy of ±1 μm in the *x*, *y* and *z* axis by a micromanipulator. A time-amplitude window discriminator (Bak Electronics, Mount Airy, MD, USA) was used to isolate single units and each accepted action potential waveform was compared with the original waveform template on a digital storage oscilloscope (Nicolet Biomedical, Madison, WI, USA). The D2 barrel column was initially identified by locating cells with less than 10 ms latency in the region of layer IV with the highest magnitude to stimulation of the D2 (principal) whisker (see Armstrong-James and Fox, 1987; Armstrong-James et al., 1992). For final selection of data only the neurons located in the D2 barrel column and confirmed histologically were included in this study.

Prior to stimulation all intact whiskers were trimmed to 10 mm lengths. Trimmed whiskers were lengthened by gluing segments of equivalent whiskers from the opposite buccal pad onto the stub with cyanoacrylate cement to establish equal lengths for controlled stimulation. Whiskers were deflected with a computer-controlled piezoelectric “bimorph” stimulator positioned with a micromanipulator just beneath or behind the whisker. Individual whiskers were deflected 300 μm forward with a 3 ms duration pulse. The principal whisker D2 and each of its immediate surround whiskers D1 and D3 were stimulated individually by presenting a block of 50 stimuli at 1 Hz to each whisker. Responses of each neuron to stimulation of three whiskers D1, D2 and D3 were recorded and stored on hard disk.

### Data analysis

A CED 1401 plus processor (Cambridge Electronic Design) and PC computer (Dell) were used to generate on-line post-stimulus time histograms (PSTHs) at 1 ms bin resolution. All data on the timing of action potentials were stored for off-line analysis. The magnitude of responses evoked from each whisker was calculated as the mean±the standard error of the mean (S.E.M.) of one block of 50 stimuli delivered at 1 Hz. The counts in each bin were adjusted for spontaneous activity by subtracting the spikes generated “*n*” ms prior to the stimulus for all forms of PSTH analysis, where *n*=equivalent period of analysis (usually 100 ms). The total number of action potentials generated 100 ms prior to the onset of the stimulus was used as the measure of spontaneous activity for each neuron. The spontaneous activity of all neurons from each layer for each group was averaged to calculate the mean spontaneous activity for different experimental groups. The significance of response change was determined by nonparametric statistical analysis using Wilcoxon matched pair sign rank (WMPSR) and Mann-Whitney *U* (MWU) tests. Latency was evaluated by median latency histogram (LH) analysis.

### Locating the recording sites

At the end of every experiment recording sites were marked by passing a DC current of 2.5 μA for 5–7 s (electrode tip positive) to produce an easily identifiable lesion roughly 50 μm diameter. The lesions were usually made at two depths along the penetration to determine the electrode path along the column. If penetrations were 100 μm apart then alternate penetrations were marked with a lesion and unmarked penetrations were determined by interpolation. For the purposes of relating neuron depth to cortical layer we placed the layer III–IV border at 450 μm and the layer IV–V border at 800 μm from the cortical surface (Li et al., 2005). The animals were perfused with 4% paraformaldehyde and the brain cryoprotected in 20% sucrose. Tangential sections of the flattened cortex were stained for cytochrome oxidase activity to locate the position of the electrode penetrations. All neurons included in the results were within the D2 barrel column, while neurons in penetrations through the septa around the barrels were excluded from the present results.

## Results

All experimental findings described below are from neurons histologically located within the D2 barrel-column. Thus, in all cases, the neurons were in the D2 barrel column and D2 was their principal whisker.

### D2-row receptive field modifications following WP

For normal non-whisker-paired (SC) rats full response profiles were available for 150 neurons in the D2-barrel column, 46 of which were in supragranular layers (SG=layers II–III), and 104 of which were in D2 barrels (layer IV). For the 15 h SCWP rats, 79 neurons were analyzed, 36 in SG layers and 43 in barrels, respectively. For EE rats, 129 neurons were analyzed, 60 in SG layers and 69 neurons in barrels. For 15 h EEWP rats, 238 neurons were available that satisfied the criteria for all analyses: 113 neurons were in the SG layers and 125 neurons were in layer IV barrels.

**Fig. 1:**
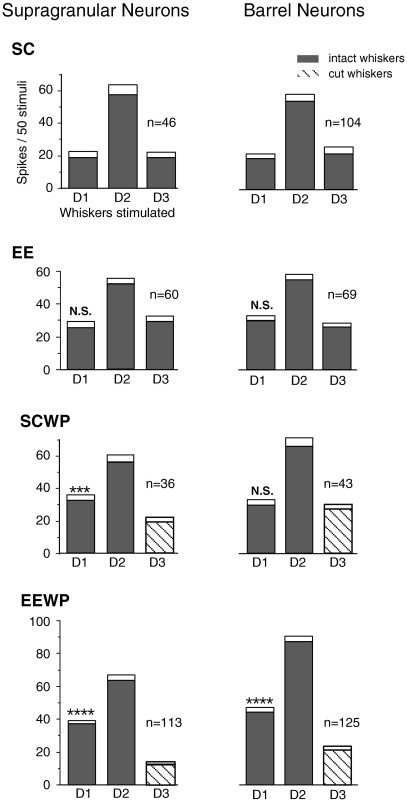
Mean response magnitudes of neurons in layers II–III (supragranular) and layer IV (barrel) to stimulation of three adjacent whiskers in the four groups of rats. All neurons were histologically confirmed to be in the D2 barrel-column in this and all other figures. Data for each neuron were collected during 100 ms PSTHs to 50 stimuli applied to each of the same three adjacent DP, D1, D2 and D3. *Left:* SG neurons; *right*: barrel neurons recorded in the same penetrations. SC: Mean response magnitudes to principal (D2) and immediate surround (D1 and D3) whiskers for rats with normal experience in standard cages. The typical control response profile is that the principal whisker generates more than twice the number of spikes than either surround whisker, and the two adjacent whiskers produce the same number of spikes in the D2 neurons in all layers. These animals had no whisker trimming. EE: Response magnitudes in rats exposed for 15 h to an EE immediately prior to neurophysiologic investigation. These animals also had no whisker trimming. SCWP: Response magnitudes in rats whisker-paired for 15 h in a standard cage. All whiskers except D1 and D2 were cut 15 h prior to neurophysiology. Note there is a significant (*P*<0.001) surround whisker bias for the SG neurons, but no increase in response to the paired whisker (D1) for barrel cells under these conditions. The dark bars represent the intact whiskers and striped bars represent cut whiskers. White caps on each bar represent S.E.M.; *n*=number of neurons in each group. Asterisks indicate that responses to D1 (paired whisker) are significantly larger than to D3 (cut whisker) within a sub-group (**** *P*<0.0001 WMPSR). Absence of asterisks indicates no significant difference between values for D1 and D3 whiskers in this and later figures. N.S., not significant. EEWP: Same as in SCWP (above) except that the animals were in the EE for 15 h followed by immediate neurophysiologic analysis. Note that a highly significant bias (greater response) has developed toward the paired whisker in both layer II–III and IV neurons.

Mean response magnitudes of neurons in the D2 barrel-column of the four groups of rats (SC, EE, SCWP and EEWP rats) to stimulation of center and immediately adjacent D-row surround whiskers are compared in Fig. 1. For all SCWP and EEWP animals the D1 whisker was left intact and paired with the uncut D2 whisker whereas D3 was the cut whisker in all cases. Layer IV barrel neuron populations (right column) are separated from SG neurons (left column).

In the normal SC group of adult rats the principal D2 whisker gives the highest magnitude response, with the D1 and D3 components of the receptive field producing lower, but equal magnitude of response (Fig. 1, top left panel), as found in previous studies on normal adult rats (see Diamond et al., 1993; Armstrong-James et al., 1994). In the EE rats, exposed to the EE without WP experience, response magnitudes to surrounds are slightly increased for all layers studied, but responses continue to remain symmetrical around the D2 whisker. In SCWP rats, the 15 h of WP fails to produce significant changes in response to surround whiskers in layer IV barrel neurons, whereas neurons in the SG already show a significant difference in response magnitude to D-row surround inputs such that the response to the paired D1 whisker was significantly greater than to cut D3 whisker (Fig. 1, 3rd row). In contrast, with the same 15 h of WP in an EE (EEWP group), mean response magnitudes to the intact surround whisker, D1, show greater than a two-fold increase over the value for the cut D3 whisker in both the supragranular and especially barrel layers (Fig. 1, lowest row).

**Fig. 2:**
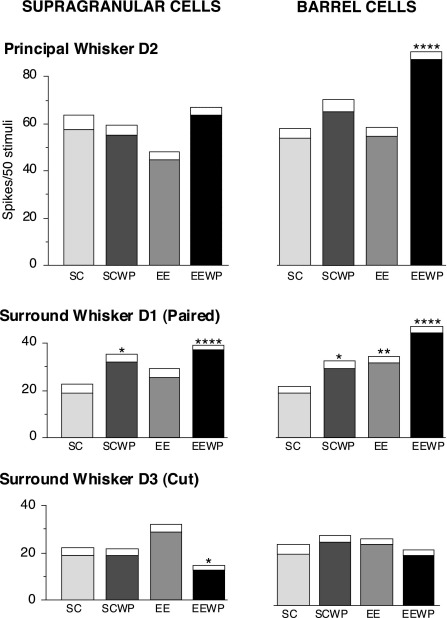
Comparisons of mean response magnitudes for neurons in the D2 barrel-column relative to the SC condition for the three DPs: principal D2 whisker, surround whisker D1 and D3. Left column: supragranular (layers II/III) neurons. *Right column*: barrel (layer IV) neurons. Response magnitudes for D1 or D3 whiskers in the whisker-paired conditions are assessed for statistical differences in comparison with the same whiskers in SCWP (* *P*<0.05, ** *P*<0.01, *** *P*<0.001, **** *P*<0.0001; MWU test). All statistical comparisons shown are between designated whisker and the same whisker for the SC condition; for further description see text. Upper row (D2): potentiation of D2 responses occurs only for barrel cells in EEWP rats relative to SC control rats after only 15 h of WP. Middle row (D1): for the D1 paired whisker, potentiation is highly significant in EEWP rats relative to SC control rats, for both supragranular and barrel neurons. Responses to D1 are significantly greater in SCWP rats than control SC rats for SG cells and barely significantly greater (*P*<0.05) than controls for barrel cells. The increased magnitude to D1 in the barrel in EEWP was much greater than for SCWP. Bottom row (D3): responses to the cut whisker D3 are significantly smaller than the same whiskers in SC for supragranular neurons only in EEWP rats. Response to D3 in barrel neurons was not significantly different from SC same whisker responses in any condition.

Aside from WP plasticity, changes in relative magnitudes of response occurred between like whiskers in the differently treated groups. Fig. 2 shows the statistical comparisons between the SC and other conditions in responses to the three D-row whiskers (DP) compared across the four conditions for the supragranular (left column) and barrel (right column) neuron populations. For EEWP animals the response magnitude to the principal whisker, D2, was very significantly greater for layer IV neurons relative to D2 response magnitude for non-whisker-paired normal SC rats (Fig. 2 upper row right: *P*<0.0001 MWU). SG neurons showed borderline differences between the non-whisker-paired normal SC rats and whisker-paired EE rats (upper row left, *P*>0.05 MWU). In SCWP rats responses to D2 whisker stimulation were not significantly different from those in SC rats in either supragranular or barrel neuron populations (*P*<0.1, MWU, in each case).

Response magnitudes of D2 neurons to stimulation of the paired D1 whisker were very significantly elevated in the EEWP group relative to SC rats (*P*<0.0001, MWU), for both supragranular and barrel neurons. In the SCWP group of animals stimulation of the paired D1 whisker shows a barely significant (<0.05, MWU) increase in magnitude following 15 h WP relative to the control non-whisker-paired SC condition for both the supragranular or barrel neuron populations. The mean response to the stimulation of the deprived D3 whisker in EEWP rats, but not SCWP rats showed a significant reduction for SG neurons relative to the same D1 whisker in normal SC rats (*P*<0.05, MWU). For barrel neurons, however, responses to the stimulation of cut whisker D3 were not significantly different in any group from corresponding responses to stimulation of D1 whisker in normal SC rats (*P*<0.1).

### Receptive field bias

**Fig. 3:**
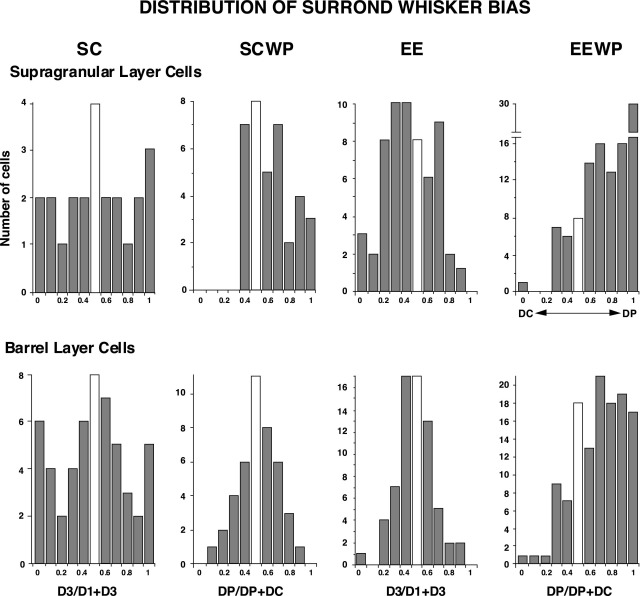
Degree of response bias to adjacent D-row surround whiskers for D2 barrel-column neurons. *Left to right*: findings are compared for SC, SCWP, EE and EEWP rats. Distributions for individual supragranular (layers II–III) neurons are in the upper row and for barrel (layer IV) neurons in the lower row. Neurons responding only to paired whisker DP (D1 in these experiments) are given a value of 1 and only a value of 0 to the cut whisker DC (D3 in these experiments) on the abscissa. No bias is a value of 0.5 (white bar). Ordinate displays the number of cells with each bias. Note that no bias occurs for untrimmed rats (SC and EE) and for layer IV neurons in SCWP rats. *Abscissa* is calculated as the sum of responses to 50 sequential stimuli to the paired whisker (DP), divided by the sum of responses to DP plus the cut whisker (DC) for each neuron.

Fig. 3 shows the relative bias of responses toward either the paired (D1) or cut (D3) whisker on a neuron by neuron basis for supragranular and barrel neurons in the four groups. In normal non-whisker-paired animals (SC) and in non-whisker-paired EE animals (EE) no bias is evident for either supragranular or barrel neurons toward responding better to either of the D-row surround whiskers. However a strong bias to the paired DP is evident for both SG neurons and barrel neurons for the EEWP condition. For SCWP rats a bias occurs at the *P*=0.01 in SG neurons, and *P*=0.02 for barrel neurons.

### Distributions of response magnitudes to surround whiskers

**Fig. 4:**
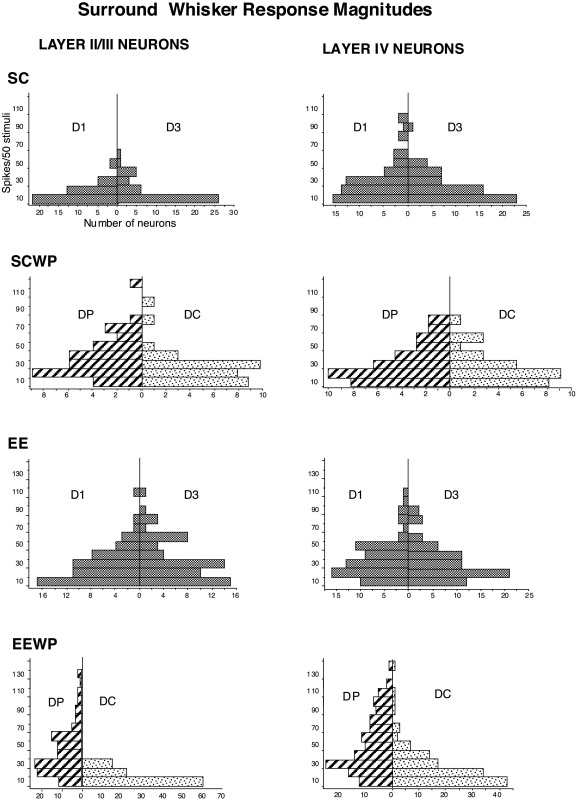
Proportionate distributions of response magnitudes to D1 and D3 surround whiskers for SC, SCWP, EE and EEWP rats. In case of SCWP and EEWP D1 is the paired whisker (DP) with high activity and D3 is the cut whisker (DC) with low activity. Note the appearance of more large magnitude responses to paired whiskers (DP), whereas the distributions are similar for cut whiskers (DC) with mostly low response magnitudes.

A quantitative estimate of plasticity is generated by examining the distributions of mean response magnitudes to cut and paired surround whiskers because it identifies the number of spikes that go into producing the activity-based response bias for neurons that respond at different levels. Fig. 4 shows these data for neurons in the four groups of rats. In the absence of WP, the distribution of response magnitudes for surround whisker responses in control SC and EE rats differed by relatively more intermediate magnitude responses (20–40 spikes/50 stimuli) occurring in all layers in EE rats relative to the control SC rats. Low magnitude responses (<10 spikes/50 stimuli) were also less common in layer IV for EE compared with SC rats. After whisker pairing the high magnitude responses (>40 spikes/50 stimuli) increased markedly in layer IV for the paired surround whisker (DP) in the EE (EEWP) but not for SCWP animals. It is noteworthy that the highest responding neurons were responsive to the active surround whisker, leaving no neurons responsive at those higher levels to the cut whisker after whisker trimming: i.e. more responsive cells were more affected.

For cut whiskers (DC) large responses (>40 spikes/50 stimuli) declined in both types of whisker paired animals (EEWP and SCWP) whereas small responses (<10 spikes/50 stimuli) were increased relative to their controls. The deprived (DC) whisker generated very few responses exceeding 40 spikes per 50 stimuli in any layer in EEWP rats. The same was the case for SCWP rats.

**Fig. 5:**
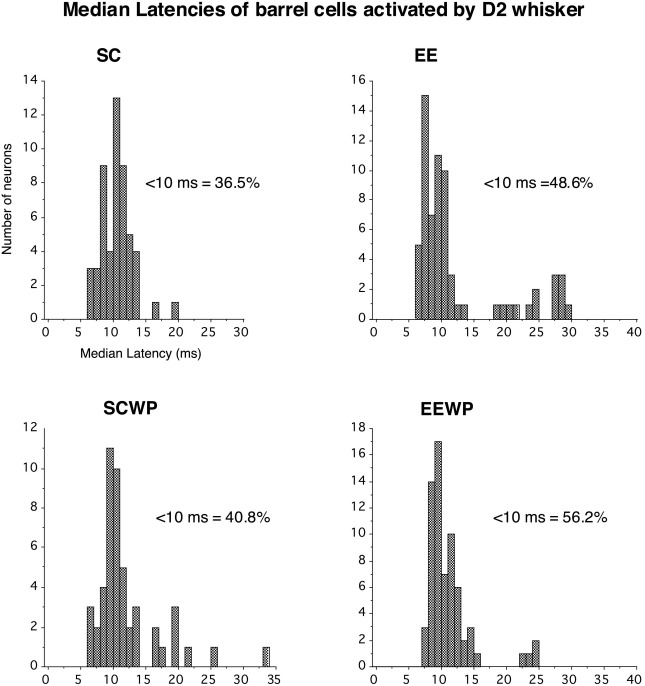
Principal (D2) whisker latencies. Distributions of median response latencies to the principal whisker for barrel neurons and supragranular neurons in the four groups of rats. Percentages indicate the proportion of responses occurring at less than 10 ms. See text for details.

In previous studies we found that latency measurements can give insights into thalamocortical vs intracortical relays contributing to principal whisker responses. Fig. 5 shows the distribution of median latencies to the D2 principal whisker for the four experimental groups. Two trends are present. First, short latency responses are more common in animals with EE experience, as witnessed by the percentage changes in responses at <10 ms latencies (insets; Fig. 5). Second for whisker-paired animals in general there is an increased incidence of shorter latencies relative to controls.

### Changes in spontaneous activity

**Fig. 6:**
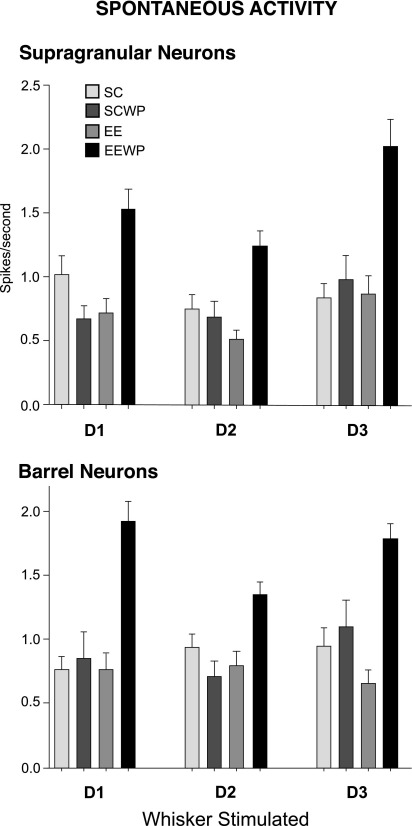
Spontaneous activity of neurons in the D2 barrel column neurons in the four groups of rats. Note the increase in spontaneous activity only in the EEWP rats that received both 15 h WP and EE experience.

Fig. 6 shows the spontaneous activity of the neurons from SG and barrel layers in the D2 barrel column. Exposure to EE by itself does not produce significant changes in the spontaneous activity (EE). On the other hand when WP for 15 h was done in EE there is significant augmentation in spontaneous discharges of neurons in both SG and barrel layers (EEWP).

## Discussion

The present study has shown that a single, roughly half-day period of exposure to a sensory and physically enriched, novel environment produces enough activity to induce plasticity in mature cortical circuits that would not occur in a standard cage. We found that response modification was accelerated and accentuated by increased environmental challenge in a large, complex three-dimensional habitat compared with that produced in a simple plastic cage. The enriched habitat generates robust plasticity in layer IV neurons as well as layers II–III neurons within a few hours, and causes responses to the paired surround whisker to double in response magnitude relative to controls. By contrast, for control animals in a standard laboratory cage, layer IV neurons (but not layers II–III neurons) failed to show significant WP plasticity within 15 h, confirming a previous study from this laboratory examining 24 h WP plasticity in standard cage adult rats (Diamond et al., 1994). Layer IV barrel cells do show significant potentiation of their principal whisker input by 7 days after the onset of whisker pairing under SCWP conditions (Armstrong-James et al., 1994).

In a previous investigation we examined the time course of WP plasticity in adult rats in the standard cage environment, finding that near-maximal surround whisker receptive field bias was generated for D2 barrel neurons after 3 days of WP (Armstrong-James et al., 1994). Near-maximal principal whisker response bias occurred after 7 days of pairing. Assuming that the degree of bias is correlated with the amount of activity, then plasticity achieved after 15 h of WP in an EE is somewhat greater than that after 7 days in the low-challenge environment of the standard cage and much more than after 3 days of WP.

### Origins of rapid plasticity

Our report documents that WP plasticity can occur within 15 h for layers II–IV in barrel cortex. On the other hand, plasticity of tactile receptive fields of some neurons in SI cortex has been reported to occur within a few minutes of altered peripheral input in adult flying fox and monkey cortex (Calford and Tweedale, 1988, 1991a,b; Byrne and Calford, 1991). However, these changes occurred in response to injury or local anesthesia rather than from changes in levels of natural sensory activity (Delacour et al., 1987; Armstrong-James et al., 1994). The mechanisms for injury-induced changes probably do not overlap completely with those induced solely by altered sensory processing and natural activity. Slowly occurring plastic changes in cortical receptive fields have been demonstrated in forepaw representation of rats following exposure to an EE for 71–113 day following weaning (Coq and Xerri, 1998) and in adult monkeys as a result of learning a tactile discrimination task over a period of weeks. Modified receptive fields were observed when the distal phalanx of three digits on one hand was placed on a flat surface that vibrated at different frequencies, only one of which was rewarded (Jenkins et al., 1990; Allard et al., 1991; Recanzone et al., 1992). These changes could characteristically require many weeks of the differential activity levels generated by the different discriminants. However, Teyler et al. (2005) recently reported LTP in the visual cortex in the form of potentiated response in the N1b component of the evoked potential. The potentiation was in response to very restricted sensory experience: a high frequency presentation of a visual checkerboard pattern that lasted for three sessions, each separated by three days.

Since the changes we observe occur within the adult brain after only a few hours of elevated sensory experience, it is more likely that modifications are at the molecular rather than a structural level. Earlier evidence suggests that morphological changes such as neurite outgrowth and number of synapses per neuron in the adult brain require several days of intense sensory experience in the absence of damage (Wallace et al., 1992). But recent evidence supports rapid changes with altered sensory experience occurring in the number of spines and synaptic contacts of cortical barrel cells (Zito and Svoboda, 2002; Trachtenberg et al., 2002; Holtmaat et al., 2005). Somatic sensory cortex WP plasticity follows Hebbian rules and can be modeled with activity-dependent algorithms (Benuskova et al., 1994) in which there is an increase in correlated firing between presynaptic inputs and postsynaptic cortical neurons that produces synaptic strengthening for the more active paired inputs. WP plasticity is similar in many ways to developmental (Hubel and Wiesel, 1970) and theoretical (Bienenstock et al., 1982) accounts of plasticity in kitten visual cortex. Analogous changes in synaptic strength occur following classical conditioning in the auditory pathways (Diamond and Weinberger, 1986; Ahissar et al., 1996) somatic sensory cortex (Delacour et al., 1987) and visual cortex (Fregnac et al., 1992). Although the evidence remains indirect, a recurring hypothesis is that at a molecular level these types of relatively fast changes depend upon NMDA receptor-dependent mechanisms in common with those for long term potentiation (LTP) and long term depression (LTD), as demonstrated so clearly in slices of hippocampus (Kandel, 1997; Lisman et al., 1997) and cortex (Bear et al., 1990).

Other combinations of activity based experience can alter the whisker representation in barrel cortex in different ways. For example, from optical measurement of blood flow in superficial barrel cortex, an expansion of the cortical representation of a single spared (D1) whisker representation in superficial cortex into adjacent deprived SG layers in adult rats has been implied. However, after 28 days of EE exposure lasting about 2 min every 3 days, it is reported that the optically assessed map shrinks relative to controls (Polley et al., 1999). In some sessions the representation of the single remaining D1 whisker fell close to zero. Cortical blood flow measurements probably relate more to the pattern of afferent excitatory and intrinsic inhibitory synaptic input than output (discharge) of cortical cells (Logothetis et al., 2001; Mathieson et al., 1998) since indirect metabolic changes are measured, but neither has been ruled out. How these interesting experiments over 28 days of highly intermittent enriched experience relate to our continuous enriched experience for 15 h is uncertain, since the paradigms and methods of measurement are very different. Further, we examined active, not deprived, barrel-column responses.

### Spontaneous activity

The mean spontaneous firing rates of neurons within the barrel column from rats kept in normal cage environments were compared with that of whisker-paired EE rats. There is some increase seen in both barrel (1.2 Hz in EEWP vs. 0.7 Hz in SC) and SG (1.4 Hz in EEWP vs. 0.9 Hz in SC). It is uncertain how spontaneous activity in neocortex impacts upon synaptic modification, since it arises from diverse sources and is highly dependent on cortical state (Armstrong-James and Fox, 1983). However, the mean level of spontaneous activity in any given cortical “state” will inevitably index mean membrane potential levels and hence excitability and a substantial role for spontaneous activity in cortical plasticity has been proposed in normal development (Katz and Shatz, 1996).

Petersen et al. (2003) found that burst firing per se in layers II–III neurons was associated with a decrease in sensory response, specifically during the burst “up” phase of depolarization. In our study we did not examine burst or stochastic firing of neurons. However, Shu et al. (2003), in studies on prefrontal or visual pyramidal neurons have shown that stochastically patterned pulses of injected current, applied to produce a moderate average increase in spontaneous activity, dramatically increase the probability of response to weak inputs, and decrease their latencies. Increased response was caused by a combination of increase in depolarization and increase in variance. Clearly a further study on experience dependent changes in spontaneous firing patterns in barrel cortex is warranted.

### Evoked activity

We have argued that the expression of short-term, 1–3 day WP plasticity of barrel cortex occurs locally at the cortical level, which is in agreement with findings on adult cat V–I plasticity (Fregnac et al., 1992; Shulz and Fregnac, 1992; McLean and Palmer, 1998). Principally, our argument is based on the observations that 1) WP plasticity is prevented by local cortical NMDAR blockade (Rema et al., 1998) and potentiation of barrel activity following aversive conditioning and whisker stimulation is reduced by partial NMDAR blockade (Jablonska et al., 1999). 2) After 15 h (present study) or 24 h (Diamond et al., 1994) of WP the layer IV thalamocortical relay neurons show no bias toward paired whiskers (SCWP condition), but responses of layers II–III neurons are modified. 3) For principal whiskers, only with much longer periods of WP do shortest latency (<10 ms), thalamocortical, monosynaptic responses show up-regulation in SC rats (Armstrong-James et al., 1994; Rema and Ebner, 1999).

With EE exposure alone, surround whisker responses are strongly increased relative to control SC animals. No overt pairing occurs with EE animals in the sense that all whiskers are similarly exposed to, and used to investigate the environment. A blanket use-dependent effect is likely in as much as greater exploration of the environment will occur in the novel environment, hence greater natural co-activation of whiskers for purposeful exploration. However this appears to be less effective than enforced use of a solitary pair of whiskers in social cage whisker-paired animals, where two barrel columns are bombarded by a high level of sensory activation whereas for other columns practically no direct inputs are being activated.

In the present study it was found that for both SCWP and EEWP rats there was a small increase in the *proportion* of short latency responses to the principal whisker with WP experience in layer IV. However, for WP experience response magnitudes to the principal whisker increased very substantially for EEWP rats; in contrast only a minor and insignificant increase occurred for SCWP rats. From this we can infer that WP experience in EE rats potentiates both short latency (presumed direct thalamo-cortical) and longer latency (presumed intracortically-relayed) responses in the principal barrel, but this does not occur in SC rats.

Short latency responses to principal whiskers in adult barrel cortex are dominated by AMPA receptor activation (Armstrong-James et al., 1993). If AMPA receptors were up-regulated in the novel, short latency responses this would be consistent with findings for expression of most forms of NMDA-dependent LTP studied in the hippocampus (Nicoll and Malenka, 1999). The principal whisker normally activates the VPM thalamic nucleus barreloids within 5–6 ms. Direct estimates on thalamocortical relay in the thalamocortical slice preparation suggest a value of 1–2 ms for most events (Agmon and Connors, 1991; Gil and Amitai, 1996) which is consistent with the observation that 30–50% of layer IV neurons respond to principal whisker stimulation within 10 ms post-stimulus in normal rats (Armstrong-James et al., 1991, 1993, 1994). Hence it would seem likely that AMPA-R as well as NMDA-R potentiation probably occurs in barrels of EE rats exposed to 15 h of WP. This would be in line with a mechanism common to many plasticity phenomena, in particular activity-induced NMDA receptor based LTP.

### Competition and inhibition

Inhibitory circuitry plays a major role in shaping the receptive fields of cortical neurons (Sillito, 1975), and this is especially true for rat somatosensory cortex (Armstrong-James and George, 1988; Simons and Carvell, 1989; Kyriazi et al., 1996). Prolonged exposure to an EE during development, causes a decrease in flat vesicle (presumed inhibitory) synapse incidence relative to round vesicle (excitatory) types in cat visual cortex synapses (Beaulieu and Cynader, 1990). The same group found that “enriched” visual cortex at maturity contains a higher proportion of orientation selective neurons and sharper orientation tuning than non-enriched cats. They suggested that higher responsivity was related to relatively lower GABAergic activity (Beaulieu and Cynader, 1990). Knott et al. (2002) showed a 36% increase in the total synaptic density within the cortical barrel of animals after a solitary intact principal whisker was oscillated intermittently for 24 h. This procedure increased both excitatory and inhibitory synapses on dendritic spines. With four days’ single whisker experience they found that the inhibitory inputs to the spines remain elevated though the synaptic density returned to pre-stimulation levels signifying there are rapid ongoing changes in the levels excitatory and inhibitory neurotransmission following short periods of altered activity. The modulation of synaptic zinc from 3 to 24 h seen by Brown and Dyck (2005) could also influence the activity of the neurons since zinc can regulate the activation of both glutamate and GABA receptor-gated ion channels.

One indicator of an acute potentiation of inhibition is the increased expression of GAD in barrels corresponding to the principal whiskers after a few days of stimulation (Welker et al., 1989). By contrast, seven days of whisker-deprivation in adult rats reduces expression of GABA-A receptors in the barrel cortex (Fuchs and Salazar, 1998), as does whisker follicle lesioning in adult mice (Skangiel-Kramska et al., 1994). Since WP experience in normal (SCWP) rats changed principal whisker responses only slightly in magnitude, it would seem that both excitatory and inhibitory relay in barrel-column neurons is raised in parallel with increased use, although in WP plasticity the excitatory drive is clearly dominant for the surround paired whisker. In this respect potentiation of excitatory inter-columnar relay between paired columns dominates over increased inhibitory drive. Cut whisker inputs might be expected to react oppositely to the paired whisker and weaken. However, there was no consistent change in response magnitude to acutely cut whiskers, except for the high activity condition of the EEWP rats (Fig. 2). Hence for normal rats, in the face of reduced intracolumnar GABAergic inhibition the loss of afferent drive by the whisker appears to be compensated by down-regulation of excitatory synapses, as in LTD. With enrichment, potentiation with brief whisker pairing of all intact inputs and reduction in response to all cut inputs occurs, in harmony with a simple rule for Hebbian interaction: whoever is most competitive gets stronger and the weaker contestant loses.

### Principal and surround paired whisker response plasticity

LTP of synaptic responses in rat barrel cortex is induced when post-synaptic depolarization or EPSPs precede single action potentials within a critical time-window of around 10–20 ms (spike timing dependent plasticity, STDP; Markram et al., 1997; Feldman 2000). Inversely, spike evocation prior to EPSP evocation lowers responses (LTD). The generation of LTP or LTD is also firing rate sensitive (Dudek and Bear 1992; Kirkwood et al., 1993). In rat V-I cortex (Sjostrom et al., 2001), firing rates, timing and cooperativity of inputs cooperate to regulate the induction of synaptic modification: higher rates of firing with greater convergence of inputs favor LTP, while LTD occurs at much lower frequencies. These factors go some way toward explaining the enhanced potentiation and increased magnitude of responses in EEWP animals, which have experienced more intense sensory experiences and show almost 2× higher spontaneous activity in barrel columns of the intact whiskers.

In the present study, EEWP animals exhibited strong potentiation of the principal whisker responses in addition to paired surround responses. No potentiation of principal whisker responses occurred in the SC animals. In previous studies on SC animals with longer periods of pairing (3–30 days), potentiation of principal whisker responses also occurred (Armstrong-James et al., 1994) although this was relatively less than for paired surround whiskers. In classical Hebbian pairing only weak (here D1 surround) inputs should be potentiated by coincident firing with strong inputs (here D2 principal) (Hebb, 1949).

One possibility for the appearance of strong D2 potentiation in EEWP animals is that the up-graded efficacy of the surround D1 surround response was sufficient to generate adequate firing coincident with enhanced subthreshold activity of synapses innervated by the D2 principal input, thus providing a “paradoxical” Hebbian potentiation of D2 input synapses. Two factors support this notion. First, in barrels and SG layers all cells act as intracortical relay cells. Every cell also has the opportunity to act as a surround activated and/or principal activated cell. When a surround input is potentiated, it will relay more activity to other connected cells. In this way cells with potentiated surrounds act as a potentiated network which can generate greater responses in target cells to principal as well as other surround inputs. With strong potentiation of surround responses we can therefore expect potentiation of disynaptic or later responses to principal inputs.

A second factor that may contribute to D2 potentiation is that spontaneous activity was raised considerably in EEWP barrels, providing more background depolarization in the D2 barrel. In turn this should facilitate coincidence of D2 EPSPS during D1 spike discharge, and hence greater probability for synaptic potentiation of D2 inputs. In cats subjected to auditory conditioning, spontaneous firing rates are elevated in reorganized areas of the tonotopic map compared with the neurons in the non-reorganized cortical regions in the same animals. Significantly, peak cross-correlation coefficients also were increased relative to the non-reorganized parts (Seki and Eggermont 2003).
